# Handedness, Grip Strength, and Memory Function: Considerations by Biological Sex

**DOI:** 10.3390/medicina55080444

**Published:** 2019-08-06

**Authors:** Paul D. Loprinzi, Joshua Franklin, Allison Farris, Seungho Ryu

**Affiliations:** Exercise & Memory Laboratory, Department of Health, Exercise Science and Recreation Management, The University of Mississippi, University, MS 38677, USA

**Keywords:** cognition, interhemispheric activation, muscular strength

## Abstract

*Background and Objective:* The objective of this study was to evaluate the potential independent and interactive effects of handedness and grip strength on episodic memory function, and whether biological sex moderated these relationships. *Materials and Methods:* 162 young adults (M_age_ = 20.7 years) completed a series of memory assessments including a subjective memory complaint evaluation and several objective measures of memory. Handedness (i.e., left-hand dominant, inconsistent handedness (ICH), and right-hand dominant) was evaluated using the Edinburgh Handedness Inventory. Handgrip strength was determined from a handgrip dynamometer. *Results:* When compared to ICH individuals, retrospective memory scores were statistically significantly worse for left-handed (*p* = 0.02) and right-handed (*p* = 0.03) individuals. Higher grip strength was statistically significantly associated with fewer retrospective memory complaints (b = 0.10, 95% CI: 0.01, 0.19, *p* = 0.04). *Conclusions:* The present study provides some suggestive evidence that ICH (inconsistent handedness) and greater grip strength are associated with fewer retrospective memory complaints. However, we did not observe any evidence of an interaction effect of handedness and grip strength on memory, and similarly, biological sex did not interact with these parameters to influence memory.

## 1. Introduction

**Figure 1:**
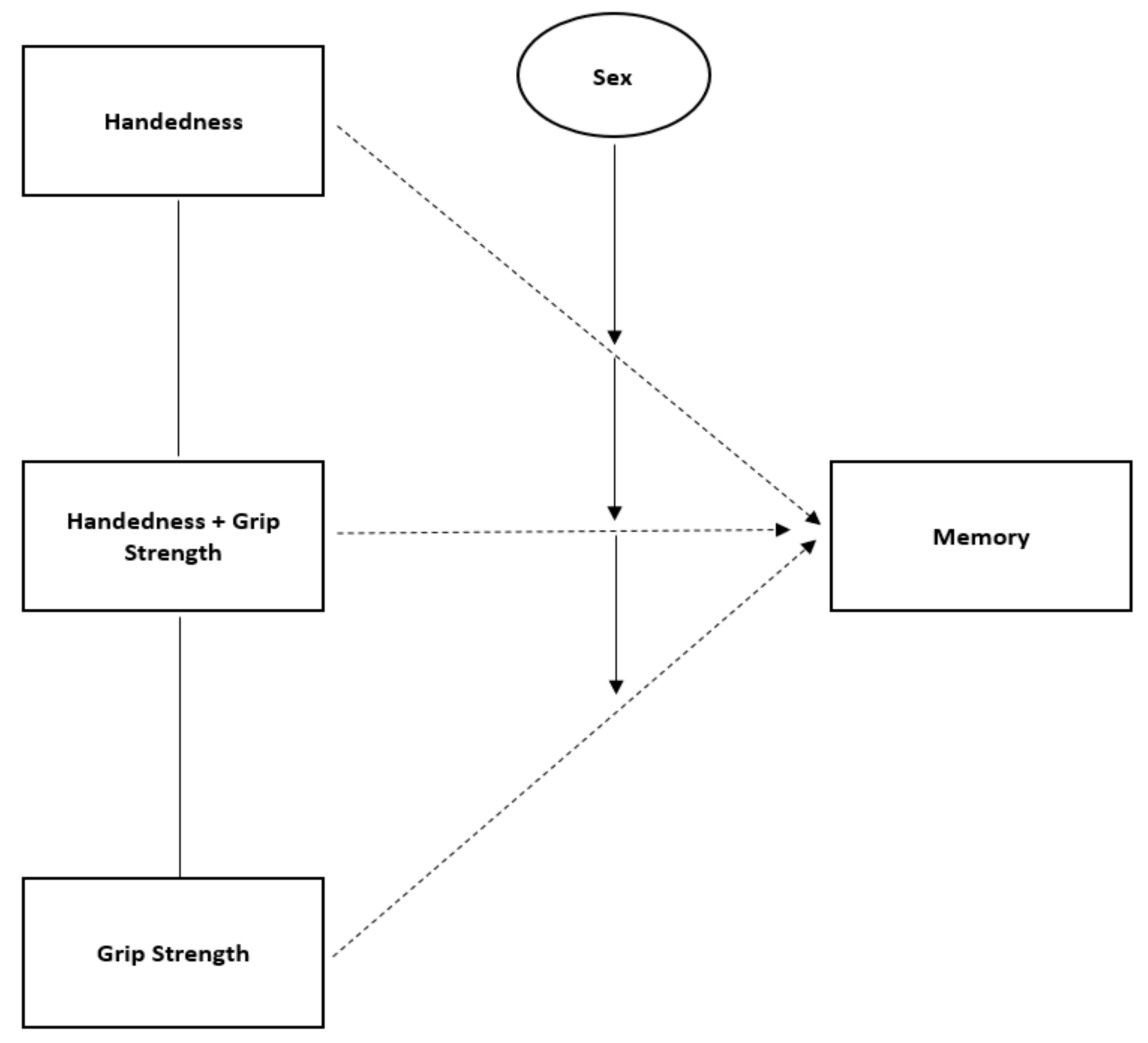
Evaluated hypothesized model suggesting potential independent and additive effects of handedness and grip strength on memory function, with moderation considerations by biological sex (i.e., whether biological sex moderates the effects of handedness on memory and grip strength on memory).

As depicted in Figure 1, the present study evaluated the potential independent and additive influence of handedness (e.g., left- and right-hand dominant, inconsistent handed) and grip strength on memory function. The following narrative will discuss these relationships, identify the gaps in the literature, and indicate the specific aims and hypotheses of the present study.

As we have discussed elsewhere [1], the degree of interhemispheric activation may play an important role in episodic memory function, or the retrospective recall of information from a spatial-temporal context. A behavioral marker of interhemispheric activation, likely mediated via the corpus callosum, includes an individual’s degree of handedness (left, right, mixed) [2,3]. Sahu et al. [4] demonstrated that inconsistent handed (ICH) individuals outperformed consistent-handers on trials 1 and 2 of a multi-trial learning word-list task, but there were no differences for trial 3 or a delayed assessment of memory. Propper et al. [5] demonstrated that ICH individuals demonstrated superior recall of both laboratory-based (word-list) and real-world autobiographical memories relative to strongly right-handed individuals. Lyle et al. [6] evaluated the effects of handedness on verbal paired associate recall, source memory, face recognition, and working memory. They demonstrated that non-strongly right-handed individuals had greater verbal paired associate recall, better source memory, but no differences were observed for face recognition or working memory (forward digit span).

Thus, there is some evidence to suggest that handedness may influence memory function. Importantly, the degree of handedness (i.e., inconsistent vs. consistent), as opposed to the directionality (i.e., right vs. left), may exert greater effects on episodic memory [7]. That is, individuals with ICH (i.e., use both their left and right hands) may have greater memory function via, for example, increased interhemispheric activation [1]. Additional work in this area is needed that more comprehensively evaluates the effects of handedness on episodic memory. Specifically, such work should consider evaluating each constituent (object, place, and time; what–where–when) of episodic memory [8], as opposed to utilizing a simple word-list task that only addresses one aspect of episodic memory (i.e., the “object” or “what” component of episodic memory).

When compared to aerobic and resistance exercise, less work, presumably, has considered the effects of grip strength on memory function. In a large sample of adults, maximal grip strength has been shown to be positively associated with visual memory, number memory, and prospective memory [9]. Although speculative, greater grip strength may, mechanistically, influence memory via associated behaviors (e.g., greater engagement in resistance exercise) and their influence on metabolic health [10]. In a recent systematic review [11] that evaluated 22 observational studies, grip strength was positively associated with global cognition as well as several cognitive subdomains including episodic memory, visuospatial ability, and working memory. Notably, however, none of the studies evaluated grip strength and hand dexterity together.

In addition to handedness, assessed via self-report, recent work has evaluated the effects of objectively measured grip strength on memory function. However, these findings are inconsistent [12,13]. For example, left handedness determined by grip strength has been shown to be associated with poorer cognitive function [12], whereas left handedness, via self-report, has been shown to be associated with better memory and attention task performance [13]. This suggests that there may be an interaction effect of handedness and grip strength on cognition.

In addition to forearm muscle size influencing grip strength [14], the dominant hand is often the hand with greater grip strength, particularly for right-hand dominant individuals, as opposed to left-hand dominant individuals [15]. This latter observation complicates the interpretation of previous work, suggesting a superior memory effect from left-handed individuals, as it is uncertain as to whether handedness, or rather, grip strength, is responsible for improvements in memory function [6]. Thus, future work should consider the potential independent and/or synergistic effects of handedness and grip strength on memory.

To our knowledge, no study has evaluated the association of both grip strength and self-reported handedness on memory function. We evaluated this among young adults, as this population is at risk of memory complaints [16] and declines in memory performance [17].

In addition to a side-to-side comparison between handedness and grip strength on memory function, another novelty of this study was to evaluate whether there was an interaction of these parameters (handedness and grip strength) on memory function. Such an interaction is plausible from several perspectives. First, if grip strength is positively associated with memory, then it is conceivable that an additive interaction effect may occur, in which those with ICH and greater grip strength may have the greatest effects on memory function. Alternatively, this situation may be less optimal for memory function. That is, those with a greater grip strength may begin closer to the peak of a Yerkes–Dodson-type inverted-U curve and being ICH may push them along the curve to a decreased performance level.

Another gap in the literature is that previous work on this topic (i.e., handedness and grip strength on memory) infrequently examines episodic memory in totality. That is, most of the work on this topic simply utilizes a word-list memory task as opposed to evaluating the individual and integrative components of episodic memory (i.e., what–where–when aspects of episodic memory). Relatedly, most of the work on this topic has focused on objective measures of memory such as objective performance on a word-list task. Much less investigated are the effects of handedness and grip strength on subjective memory function (i.e., an individual’s perception of their memory functioning). This is important, as although some research has demonstrated concordance between subjective and objective measures of memory function [18,19], other research has demonstrated a dissociation between these assessments (with this dissociation moderated by cognitive function status [20]) [21]. This latter observation either suggests that subjective measures of memory inadequately represent actual memory function, or, alternatively, it is possible that subjective and objective measures of memory may represent distinct memory systems. For example, memory questionnaires have been shown to be associated with autobiographical memory vividness and not the number of internal (episodic) details [22].

Our first aim was to evaluate whether handedness was directly associated with subjective and objective (including what–where–when aspects of episodic memory) memory function. We hypothesized that ICH individuals would have greater overall memory function (better objective memory performance and fewer subjective memory complaints), presumably through enhanced interhemispheric activation.

Our second aim was to evaluate whether grip strength was directly associated with subjective and objective memory function. We also hypothesized that those with greater grip strength would have greater memory function.

Our third aim was to evaluate whether handedness and grip strength interacted to influence memory function. We hypothesized that an interaction would be present, and specifically, that ICH individuals with greater grip strength would have the greatest memory performance.

Finally, our fourth aim was to evaluate whether these above-listed objectives were influenced by biological sex, which is plausible given that biological sex seems to play a critical role in influencing episodic memory [23]. As we have thoroughly discussed elsewhere [23], biological sex is an important contributor to memory function. Females tend to outperform males in most memory tasks [23]. However, this is a complex dynamic, as recent research suggests that sex may interact with handedness on cognition, with reduced brain lateralization in women explaining lower cognition in select cognitive parameters [24]. Relatedly, in a large cross-sectional study of older adults and based on grip strength, left-handed women scored worse than right-handed women in immediate and delayed memory [12]. Such an effect was not observed for men [12]. Adding further complexity to this, past work has shown that right-handed males and left-handed females performed better in visuospatial cognitive tasks, whereas left-handed males and right-handed females performed better in verbal-related cognition [25]. Conversely, Sahu et al. [4] did not demonstrate a sex by handedness interaction on a word-list memory task.

## 2. Materials and Methods

### 2.1. Study Design and Participants

Participants completed a single laboratory visit. During this visit, participants completed a computerized episodic memory task, which is a survey assessing subjective memory complaints and handedness, and had their grip strength objectively measured. Details of these assessments are described below. In total, 162 participants completed the study, which occurred between January and May 2019. All data collection occurred in the Exercise and Memory Laboratory at the University of Mississippi. This study was approved by the Ethics Committee at the University of Mississippi (#19-069). All participants provided written, informed consent prior to participation. Due to potential confounding effects on memory, participants were excluded if they:

- Self-reported as a daily smoker [26,27]
- Self-reported being pregnant [28]
- Exercised within 5 h of testing [29]
- Consumed caffeine within 3 h of testing [30]
- Had a concussion or head trauma within the past 30 days [31]
- Took marijuana or other mind-altering drugs within the past 30 days [32]
- Were considered a daily alcohol user (>30 drinks/month for women; >60 drinks/month for men) or consumed alcohol on the day of testing [33]

### 2.2. Study Protocol

**Scheme 1:**
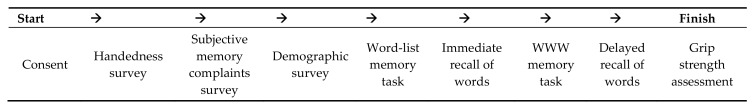
Study protocol.

Study protocol is presented in Scheme 1.

### 2.3. Demographic Survey

Participant characteristics including age, gender, and race-ethnicity were self-reported. At this time, height and weight were objectively measured. Furthermore, at this time, participants self-reported whether they engaged in resistance exercise at least twice a week and reported the amount of time per week they engaged in moderate-to-vigorous physical activity (Physical Activity Vital Signs survey).

### 2.4. Handedness Assessment

Handedness was assessed from the Edinburgh Handedness Inventory – Short Form, which has demonstrated evidence of validity [34]. Participants were asked to indicate their preferences in the use of hands in the following activities or objects: writing, throwing, toothbrush, and spoon. Responses options included: always right, usually right, both equally, usually left, and always left. For each item, the following scores were given: 100 (always right), 50 (usually right), 0 (both equally), −50 (usually left), and −100 (always left). The Laterality Quotient was calculated by summing these four scores and then dividing it by four. Handedness classification was determined from the following Laterality Quotient scores: −100 to −61 (left-handed), −60 to 60 (mixed handed; ICH), and 61 to 100 (right-handed) [34].

### 2.5. Grip Strength Assessment

Grip strength (kg) was assessed using a Takei digital grip strength dynamometer (Model T.K.K. 5401). Similar to other protocols [35], with the arm parallel to the side of the body, testing was performed in a standing position. Participants were instructed to squeeze the dynamometer as hard as possible while exhaling. Tests were completed on both hands, with each hand tested three times, alternating hands between trials with a 60 s recovery period between trials. The outcome metric was the highest grip strength across the six trials.

### 2.6. Memory Assessment

Word-List Task. The word list was composed of 15 words selected from the MRC Psycholinguistic Database [36]. All words were nouns, had concreteness, imageability, and familiarity ratings between 530 and 700, and included 5–10 letters and 1–2 syllables. Participants were exposed to 15 words, one word at a time on a computer screen for three seconds. Participants were exposed to two cycles of this and then completed a free recall of the words (immediate memory recall). Participants then engaged in the WWW task, which took approximately 10 minutes to complete. A delayed free recall of the words occurred after the WWW task. Notably, other related word-list memory tasks have demonstrated evidence of validity and reliability [37]. The outcomes for this word-list memory task included the immediate and delayed recall performances (number of words recalled).

**Figure 2:**
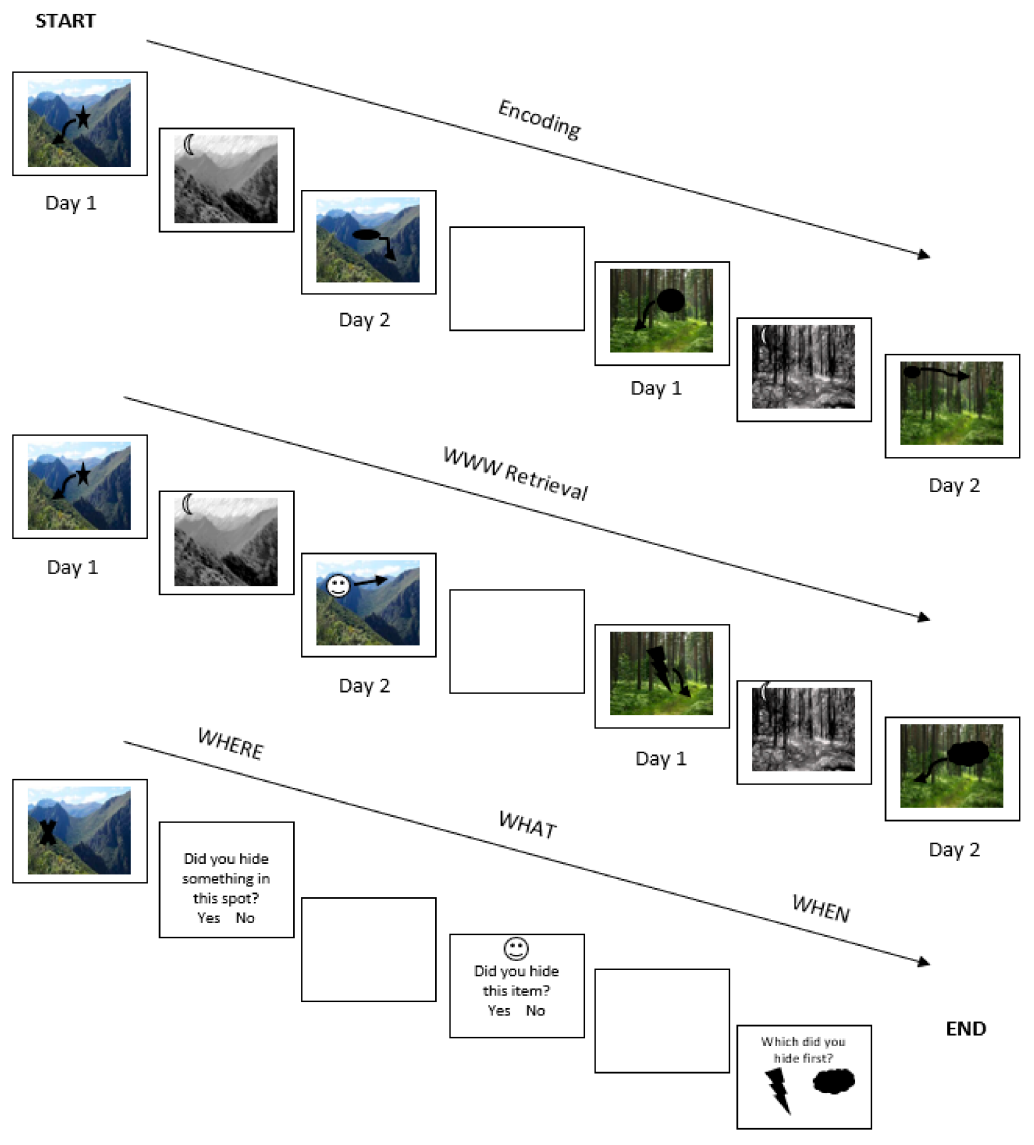
Illustrative example of the episodic memory task employed in our study. Items were hidden in two different scenes, across two days labeled as “day 1” and “day 2” (virtual passage of time). Following this encoding portion, participants were then instructed to indicate where they hid each item for both “day 1” and “day 2” and for each scene (WWW score). After this, participants completed the “where”, “what”, and “when” parameters by the recognition and discrimination of the items displayed.

WWW Task. The WWW task (What–Where–When) is a computerized task assessing ‘what–where–when’ episodic memory that takes approximately 10 minutes to complete. Details of this task have been discussed elsewhere [38,39,40]. A schematic of this task is shown in Figure 2. In brief, this task involves ‘hiding’ items in various scenes, then later indicating what items were hidden, where, and on what occasion. This requires the integration of item, location, and temporal memory into a single coherent representation (What–Where–When memory, WWW). Participants were also assessed for their memory of the individual components (what, where, and when) without requirement for integration. Internal consistency for these tasks has been previously demonstrated (ICCs > 0.7) [39]. The outcome variables assessed included an absolute WWW score (where the location of the correct object for the correct time was identified exactly), and the proportion of correct responses for the separate what, where, and when sub-tasks. This study used the ‘medium’ difficulty version of the task and assessed 16 unique item–location–time combinations.

### 2.7. Subjective Memory Complaints Assessment

Subjective memory complaints were assessed using the Prospective–Retrospective Memory Questionnaire [41,42,43]. This questionnaire involves 16 items (eight prospective and eight retrospective). An example item for prospective memory complaints includes “*Do you decide to do something in a few minutes’ time and then forget to do it*?” An example item for retrospective memory complaints is “*Do you fail to recall things that have happened to you in the last few days*?” For all items, response options include very often (1), quite often (2), sometimes (3), rarely (4), and never (5). Responses from the eight prospective and eight retrospective items were summed (range, 8–40), with higher values indicative of fewer subjective memory complaints. This subjective memory complaint questionnaire has previously demonstrated evidence of reliability and validity [44,45,46]. For the present sample, the overall internal consistency, as measured by Cronbach’s alpha, was 0.88. For the present sample, the Cronbach’s alpha was 0.79 and 0.80, for prospective and retrospective memory complaints, respectively. 

### 2.8. Statistical Analysis

All statistical analyses were computed in JASP (v. 0.9.2, Amsterdam, The Netherlands). Statistical significance was established as a nominal alpha of 0.05. Partial eta-square was calculated as an estimate of effect size (η^2^_p_). The analytical approach for each of the study aims are shown below. Assumptions for the analytical tests (e.g., normality, collinearity) were checked and were found not to be violated.

Aim 1: Evaluate whether handedness is directly associated with subjective and objective (including what–where–when aspects of episodic memory) memory function.

Analysis 1: Multivariable linear regression was used to evaluate the association between handedness and memory function. Separate models were computed for each episodic memory outcome. In each model, covariates included age, sex, and measured BMI (body mass index; kg/m^2^).

Aim 2: Evaluate whether grip strength is directly associated with subjective and objective memory function.

Analysis 2: Multivariable linear regression was used to evaluate the association between grip strength and memory function. Separate models were computed for each episodic memory outcome. In each model, covariates included age, sex, and measured BMI (kg/m^2^).

Aim 3: Evaluate whether handedness and grip strength interact to influence memory function.

Analysis 3: Interaction models were evaluated by creating a cross-product term of handedness and grip strength (laterality quotient * grip strength) and included this cross-product term, along with the main effect terms and covariates (age, sex, and measured BMI) in a linear regression model.

Aim 4: Evaluate whether handedness and biological sex as well as grip strength and biological sex interact to influence memory function.

Analysis 4: Interaction models were evaluated by creating a cross-product term of handedness and sex (laterality quotient * sex) and included this cross-product term, along with the main effect terms and covariates in a linear regression model. Similarly, interaction models were evaluated by creating a cross-product term of grip strength and sex (grip strength * sex) and included this cross-product term, along with the main effect terms and covariates in a linear regression model. Finally, a three-way interaction term (laterality quotient * grip strength * sex) was created and included in a linear regression, along with these three main effect terms and covariates.

## 3. Results

Study Variable Characteristics. Characteristics of the study variables are shown in Table 1. Participants on average were 20.7 (1.1) years of age, 69.1% female, 77.2% non-Hispanic white, and had a mean measured BMI of 24.4 (4.5) kg/m^2^. One-third (34%) of the sample engaged in resistance training at least two days per week. Notably, the results were not different when we statistically controlled our main analyses by the resistance training status. When compared to left-hand dominant (10.5%) and ICH (5.5%) individuals, the majority (84.0%) of the sample were right-hand dominant.

Handedness by Grip Strength. Table 2 displays the average left- and right-hand grip strength scores across the handedness classifications (left-hand dominant, ICH, and right-hand dominant). There was not a significant main effect for grip strength (F = 2.07, *p* = 0.15, η^2^_p_ = 0.01) or handedness (F = 1.27, *p* = 0.28, η^2^_p_ = 0.02), but there was a grip strength by handedness interaction (F = 8.14, *p* < 0.001, η^2^_p_ = 0.09). Right-hand dominant individuals, on average, had a higher right grip strength (33.2 kg) when compared to their left grip strength (31.0 kg) (*p* < 0.001). This, however, was not the case for left-hand dominant individuals as their right grip strength (35.4 kg) was not different from their left grip strength (36.4 kg) (*p* = 0.26). Grip strength also did not differ for ICH individuals (*p* = 0.76).

Aim 1: Evaluate whether handedness is directly associated with subjective and objective memory function.

Episodic memory scores across handedness classification are shown in Table 3. The only model that approached statistical significance was for retrospective memory complaints. Among those who were classified as left-handed, ICH, and right-handed, respectively, the mean (SD) retrospective subjective memory complaint scores were 27.8 (5.0), 32.3 (3.6), and 28.9 (4.7) (F(2,159) = 2.74, *p* = 0.06, η^2^_p_ = 0.03). When compared to ICH individuals, retrospective scores were statistically significantly worse for left-handed (*p* = 0.02) and right-handed (*p* = 0.03) individuals. For both retrospective (*p* = 0.32) and prospective (*p* = 0.75) memory complaints, there were no differences between left- and right-handed individuals.

Given the limited number of individuals who were classified as left-handed or ICH, further analyses were computed that treated handedness as a continuous variable (i.e., laterality quotient). These results, via linear regression analyses, are shown in Table 4. These results were similar to the findings when handedness was expressed as a categorical variable (Table 3). None of the linear regression models were statistically significant, in that handedness was not associated with memory function for any of the models (Table 4).

Aim 2: Evaluate whether grip strength is directly associated with subjective and objective memory function.

The results regarding the association between grip strength and memory function are shown in Table 5. Grip strength was statistically significantly associated with retrospective memory complaints (b = 0.10, 95% CI: 0.01, 0.19, *p* = 0.04). That is, those with a higher grip strength had fewer retrospective memory complaints. This association remained statistically significant even when further adjusting for self-reported habitual engagement in moderate-to-vigorous physical activity (min/week) and whether they engaged in resistance training at least two times per week (yes/no) (b = 0.10, 95% CI: 0.002, 0.20, *p* = 0.04). Similarly, even when adding handedness (laterality quotient) to this latest adjusted model, grip strength remained statistically significantly associated with retrospective memory (b = 0.10, 95% CI: 0.001, 0.20, *p* = 0.04).

The results also approached statistical significance for prospective memory complaints (b = 0.08, 95% CI: −0.01, 0.17, *p* = 0.07). No other models were statistically significant. Results were similar across additional sensitivity models (results not shown) that utilized a different grip strength metric (e.g., instead of the highest grip strength value, we utilized average grip strength across both hands, just the left hand, and just the right hand).

Aim 3: Evaluate whether handedness and grip strength interact to influence memory function.

There was no evidence of an interaction effect of handedness and grip strength on prospective memory complaints (*p* = 0.97), retrospective memory complaints (*p* = 0.87), overall WWW (*p* = 0.47), what-loop (*p* = 0.54), where-loop (*p* = 0.23), when-loop (*p* = 0.31), immediate word-list recall (*p* = 0.36), and delayed word-list recall (*p* = 0.10).

Aim 4: Evaluate whether handedness and biological sex as well as grip strength and biological sex interact to influence memory function.

There was no evidence of an interaction effect of handedness and sex on prospective memory complaints (*p* = 0.96), retrospective memory complaints (*p* = 0.73), overall WWW (*p* = 0.53), what-loop (*p* = 0.44), where-loop (*p* = 0.97), when-loop (*p* = 0.80), immediate word-list recall (*p* = 0.93), and delayed word-list recall (*p* = 0.61). Interaction models were also evaluated for biological sex and grip strength on memory function, and the results were similar (i.e., no significant interaction models). Similarly, there were no statistically significant three-way interactions (laterality quotient * grip strength * sex) on any of the memory outcomes.

## 4. Discussion

The aims of this investigation were multifold, but focused on the potential independent and additive influences of handedness and grip strength on episodic memory function. Our results were in partial alignment with our hypotheses. We demonstrated that ICH and higher grip strength were associated with better memory function (fewer retrospective memory complaints).

Although ICH individuals had fewer retrospective memory complaints, it is important to not overemphasize this relationship for several reasons. First, we were not able to replicate this handedness–memory relationship when handedness was expressed as a continuous variable (i.e., laterality quotient). Furthermore, very few participants in our sample were classified as ICH; thus, this observed relationship may not be a reliable relationship. Despite these concerns, this observation did align with other research showing that the degree of handedness, as opposed to the directionality, may exert greater effects on episodic memory [7]. As per the Hemispheric Encoding and Retrieval and Asymmetry (HERA) model [47], encoding may predominate in the left hemisphere, with memory retrieval primarily occurring in the right hemisphere. This, however, may be contingent upon the memory task and complexity of the task [1]. Mix-handed individuals are thought to have increased interhemispheric activation, which in turn, may facilitate the encoding-retrieval relationship [7]. Moreover, ICH individuals may have a larger corpus callosum and greater functional access to the right hemispheric processing [48].

Another notable finding of our study that deserves discussion is the observation that grip strength was associated with fewer retrospective memory complaints. Admittedly, this is a difficult observation to fully understand. Although other research demonstrates that grip strength is associated with better overall cognition as well as greater memory function [11], it is uncertain as to whether grip strength directly influences memory, or rather, whether grip strength serves as a proxy measure for other situations that drive this relationship. As an example, and as one might perceive, greater grip strength may result from more habitual engagement in resistance training. However, traditional resistance training does not appear to appreciably alter grip strength (discussed in detail elsewhere [49]) [50]. Alternatively, perhaps grip strength is a marker for overall health (e.g., metabolic health), which in turn, may be associated with greater memory function [10]. Although interesting, clearly the relationship between grip strength and health is difficult to explain.

The present study also demonstrated some evidence to suggest that handedness and grip strength are associated with fewer subjective memory complaints, particularly retrospective memory complaints. We did not, however, observe any associations with any of the objective measures of memory. Although other research, at times, has demonstrated a dissociation between subjective and objective memory function [20,21], it is not clear as to why handedness and grip strength may be associated with subjective memory over objective memory function. Although speculative, perhaps subjective memory complaints are more enduring, and thus, is a more reliable phenomenon, whereas objective memory performance may be quite variable [51] and vulnerable to various factors such as the time of day in which it is assessed.

Limitations from this study to consider include the following. First, it was not logistically possible to test all of the participants at the same time of day, in order to prevent a time-of-day effect. In fact, this is unlikely to be an optimal strategy as the peak and off-peak memory times may vary by person [52]. Second, as with most handedness studies, relatively few participants were left-hand dominant or ICH, and as such, future research should consider over sampling these individuals to induce more reliable estimates. Third, the instrument used to evaluate handedness included a wide range for the classification of ICH (i.e., −60 to 60), and as such, these ICH individuals may have varied degrees of ICH. This is likely to have an influence on the degree of interhemispheric activation potentially induced by ICH. Furthermore, there may also be between-subject heterogeneity in the degree of intrahemispheric activation across the different levels of handedness, which should be more carefully considered in future research. Relatedly, the handedness survey that we employed did not take into consideration the degree of coordinated activity between the hands (e.g., one hand completing one task while the other hand completing another task), which may uniquely influence interhemispheric activation. The strengths of this study are multifold and include the study’s novelty, relatively large overall sample size, the inclusion of assessments of both handedness and grip strength, and the incorporation of both subjective and objective measures of memory function.

## 5. Conclusions

In conclusion, the present study provides some suggestive evidence that ICH (inconsistent handedness) and greater grip strength are associated with fewer retrospective memory complaints. However, we did not observe any evidence of an interaction effect of handedness and grip strength on memory, and similarly, biological sex did not interact with these parameters to influence memory.

## Figures and Tables

**Table 1:** Study variable characteristics (N = 162).

Variable	Point Estimate	SD
**Demographics**		
Age, mean years	20.7	1.1
Gender, % Female	69.1	
Race-Ethnicity, % White	77.2	
BMI, mean kg/m^2^	24.4	4.5
**Behavioral**		
Resistance Training 2x/week, % Yes	34.0	
MVPA, mean min/week	166.8	127.8
**Handedness**		
Laterality Quotient, mean	70.2	62.0
Classification, %		
Left-hand dominant	10.5	
Inconsistent handedness	5.5	
Right-hand dominant	84.0	
**Grip Strength**		
Left-hand, mean kg	31.5	10.3
Right-hand, mean kg	33.3	10.1
Average of both hands kg	32.4	10.1
Highest value, mean kg	36.2	10.9
**Subjective Memory**		
Prospective Complaints, mean	26.7	4.5
Retrospective Complaints, mean	29.0	4.8
**Objective Memory**		
Word-List Recall		
Immediate Memory, mean # words	9.3	2.1
Delayed Memory, mean # words	7.6	2.4
WWW Task		
Overall, mean %	47.2	21.9
What-Loop, mean %	96.8	7.3
Where-Loop, mean %	86.5	11.7
When-Loop, mean %	92.9	8.4

BMI, Body Mass Index; MVPA, Moderate-to-Vigorous Physical Activity; #, Number.

**Table 2:** Hand grip strength based on handedness.

Grip Strength	Left-Hand Dominant	Inconsistent Handed	Right-Hand Dominant	Test-Statistic
Left-hand, mean kg	36.4 (12.0)	30.1 (5.7)	31.0 (10.2)	F(grip) = 2.07, *p* = 0.15, η^2^_p_ = 0.01 F(hand) = 1.27, *p* = 0.28, η^2^_p_ = 0.02 F(grip × hand) = 8.14, *p* < 0.001, η^2^_p_ = 0.09
Right-hand, mean kg	35.4 (11.8)	30.7 (4.5)	33.2 (10.1)	

Values in parentheses represent standard deviations. A 2 (grip; left vs. right) × 3 (hand classification) RM-ANOVA was computed.

**Table 3:** Memory performance (mean/sd) across handedness classification.

Word-List Memory	Left-Handed	Inconsistent Handed	Right-Handed	Test-Statistic
Immediate Memory Word List	9.5 (1.9)	10.1 (1.5)	9.2 (2.2)	F(2,159) = 0.69, *p* = 0.49, η^2^_p_ = 0.01
Delayed Memory Word List	7.5 (2.5)	8.9 (2.8)	7.6 (2.4)	F(2,159) = 1.20, *p* = 0.30, η^2^_p_ = 0.01
**WWW Memory Task**				
Overall WWW	44.9 (17.8)	40.8 (28.3)	47.8 (22.0)	F(2,159) = 0.53, *p* = 0.58, η^2^_p_ = 0.01
WWW What-Loop	97.7 (5.1)	97.9 (3.1)	96.6 (7.7)	F(2,159) = 0.29, *p* = 0.74, η^2^_p_ = 0.01
WWW Where-Loop	83.4 (12.9)	83.4 (13.7)	87.0 (11.4)	F(2,159) = 1.00, *p* = 0.36, η^2^_p_ = 0.01
WWW When-Loop	93.2 (7.1)	92.6 (5.3)	92.9 (8.7)	F(2,159) = 0.01, *p* = 0.98, η^2^_p_ = 0.01
**Subjective Memory Complaints**				
Prospective	26.6 (3.6)	28.4 (4.4)	26.6 (4.6)	F(2,159) = 0.67, *p* = 0.51, η^2^_p_ = 0.01
Retrospective	27.8 (5.0)	32.3 (3.6)	28.9 (4.7)	F(2,159) = 2.74, *p* = 0.06, η^2^_p_ = 0.03

**Table 4:** Association between handedness (IV) and memory function (DV).

Model	b	95% CI	*p*-Value
**Subjective Memory Complaints**			
Laterality Quotient → Prospective Memory Complaints	0.001	−0.01, 0.01	0.79
Laterality Quotient → Retrospective Memory Complaints	0.001	−0.01, 0.01	0.89
**Objective Memory Function**			
Laterality Quotient → Immediate Memory Word List	−0.002	−0.008, 0.003	0.42
Laterality Quotient → Delayed Memory Word List	−0.002	−0.007, 0.005	0.79
Laterality Quotient → Overall WWW	0.03	−0.01, 0.09	0.20
Laterality Quotient → WWW What-Loop	−0.007	−0.02, 0.01	0.44
Laterality Quotient → WWW Where-Loop	0.02	−0.009, 0.05	0.16
Laterality Quotient → WWW When-Loop	0.005	−0.01, 0.02	0.66

Each model was statistically controlled for age, sex, and BMI. b, unstandardized regression coefficient.

**Table 5:** Association between grip strength (IV) and memory function (DV).

Model	b	95% CI	*p*-Value
**Subjective Memory Complaints**			
Grip Strength → Prospective Memory Complaints	0.08	−0.01, 0.17	0.07
Grip Strength → Retrospective Memory Complaints	0.10	0.01, 0.19	0.04
**Objective Memory Function**			
Grip Strength → Immediate Memory Word List	0.02	−0.02, 0.06	0.41
Grip Strength → Delayed Memory Word List	0.008	−0.04, 0.05	0.76
Grip Strength → Overall WWW	−0.08	−0.53, 0.36	0.70
Grip Strength → WWW What-Loop	0.11	−0.03, 0.26	0.14
Grip Strength → WWW Where-Loop	−0.03	−0.28, 0.21	0.78
Grip Strength → WWW When-Loop	−0.15	−0.32, 0.01	0.08

Each model was statistically controlled for age, sex, and BMI. The highest grip strength value from the participant was used as the grip strength variable. b, unstandardized regression coefficient.
